# Improving the Evidence Base for Trauma Care: Progress in the International CRASH-2 Trial

**DOI:** 10.1371/journal.pctr.0010030

**Published:** 2006-10-27

**Authors:** The CRASH-2 Trial Collaborators

There is an urgent need to improve the evidence base for trauma care. Large-scale clinical trials can provide important answers, but they depend on effective international collaboration. The purpose of this article is to bring one such trial, the CRASH-2 trial, to the attention of physicians around the world. The CRASH-2 trial, which is currently under way (registered at ISRCTN86750102), is a large placebo-controlled trial being performed in trauma patients at risk of significant haemorrhage. The trial examines the effects of antifibrinolytic treatment on death and the need for blood transfusion. If such a simple and widely practicable treatment as the antifibrinolytic agent tranexamic acid (TXA) were shown to reduce blood loss after trauma, thousands of trauma deaths could be preventable worldwide. Furthermore, large numbers of patients would avoid the risks of blood transfusion. The trial is now recruiting and has already randomised more than 2,000 trauma patients. In this article, we present an overview of the progress of the trial and urge physicians working in emergency care to consider joining this international collaborative effort.

## Background to the Trial

Between the ages of 5 and 45 years, trauma is second only to HIV/AIDS as a cause of death. Each year, worldwide, more than 3 million people die as a result of trauma, many after reaching hospital [1]. Among trauma patients who do survive to reach hospital, excessive blood loss is a common cause of death, accounting for nearly half of in-hospital trauma deaths [2]. Central nervous system injury and multi-organ failure account for most of the rest, both of which can be worsened by severe bleeding [3]. It is well documented that funding for trauma research is less than for almost any other cause of human suffering. A World Health Organization study compared the level of research funding with current and projected (2020) disease burden. The results clearly showed the relative under-funding of research on injuries. A more recent study from the United Kingdom shows that injury remains the most under-funded of all health issues [4,5].

Systemic antifibrinolytic agents are widely used in major surgery to reduce surgical blood loss. A recent systematic review [6] of randomised controlled trials of antifibrinolytic agents (mainly aprotinin or TXA) in elective surgical patients identified 89 trials including 8,580 randomised patients. The results of the systematic review showed that these treatments reduced the numbers needing transfusion by one-third, reduced the volume needed per transfusion by one unit, and halved the need for further surgery to control bleeding (all differences were statistically significant).

We hypothesised that since the haemostatic changes after injury are similar to those after surgery, it is important to find out whether antifibrinolytic agents similarly reduce blood loss, the need for transfusion, and mortality following trauma. The CRASH-2 (clinical randomisation of an antifibrinolytic in significant haemorrhage [7]) trial was designed to test precisely this hypothesis. Once complete, this trial will, to our knowledge, be one of the largest clinical trials in trauma ever conducted.

The trial aims to recruit 20,000 patients worldwide, and recruitment began in May 2005. Recruitment is expected to be complete in December 2009. At the time of this writing, 2,200 patients have been recruited and the CRASH-2 trial is already the largest clinical trial among patients following trauma with significant ongoing haemorrhage.

## Overview of Trial Design

CRASH-2 is a randomised, double-blind placebo-controlled trial comparing TXA with matching placebo (saline), the details of which have been published elsewhere [7]. TXA is the antifibrinolytic of choice because it is much less expensive than others within the drug category, has been used for many years, and is a simple synthetic molecule that does not require a test dose and has less risk of allergic reactions. The dosing regimen is a loading dose of 1 g of TXA or placebo in a 100-ml infusion over 10 minutes followed by a maintenance infusion of 120 mg/hour for 8 hours. In the emergency situation, the administration of a fixed dose is more practicable because weighing patients in such situations is difficult. Therefore, a dose within the dose range that has been shown to inhibit fibrinolysis and provide clinical benefit was selected for CRASH-2.

All adult trauma patients who are within 8 hours of their injury and have either significant haemorrhage (systolic blood pressure less than 90 mm Hg and/or heart rate more than 110 beats per minute), or who are considered to be at risk of significant haemorrhage, are eligible for trial entry if they appear to be at least 16 years old. Although entry is allowed for up to 8 hours after injury, the earlier that patients can be treated the better. There are no other pre-specified exclusion criteria, as the fundamental eligibility criterion is the responsible physician's “uncertainty” about using TXA in a particular adult with traumatic haemorrhage. The use of simple entry criteria and the uncertainty principle, as is recommended in the context of large-scale trials, allows participating physicians to use their clinical judgement when deciding whether or not to enroll patients in the trial, just as they do in normal medical practice. This is particularly appropriate in the context of traumatic haemorrhage, in which it is necessary to evaluate a range of clinical signs (also taking into account remedial measures such as fluid resuscitation) when establishing the presence or absence of major haemorrhage.

## Obtaining Consent in Emergency Situations

This trial involves a population likely to have impaired consciousness due to their injury, and situations where randomisation to treatment has to be completed in the context of a clinical emergency. The legal requirement for consent in this population has made it difficult to conduct the trial in some countries, especially those that require prior consent from the patient or a legally identified representative. All patients are randomised according to consent procedures that adhere to the legal and ethical requirements of each country and in accordance with International Conference on Harmonisation Good Clinical Practice E6 guidelines, section 4.8.15 [8].

## Randomisation

Patients are randomised in one of two ways. Hospitals with reliable telephone access through which the recruiting physicians are able to provide baseline data in English use the central telephone randomisation service provided by the Clinical Trial Service Unit (CTSU) in Oxford. During the call, which lasts 2–3 minutes, baseline data are collected and recorded on the central computer. To achieve a reasonable balance on the key prognostic factors, the allocation used a minimisation algorithm, balancing for sex, age (16–24 years, 25–34 years, 35 years and older), hours since injury (1 hour or less, 1–3 hours, more than 3 hours), type of injury (blunt or penetrating), Glasgow Coma Scale (3, 4–5, 6–8, 9–12, 13–15), systolic blood pressure (>89, 76–89, 50–75, 1–49, 0), respiratory rate (>29, 10–29, 6–9, 1–5, 0), central capillary refill time (2 or less, 3–4, 5 or more) and bearing in mind what packs remain at that hospital. The allocated treatment pack number is then given and recorded on the trial entry form. In hospitals in which physicians are unable to use central randomisation, a local pack system is used. At such hospitals, baseline information is collected on the trial entry form and the next consecutively numbered treatment pack is taken from a box of eight packs.

## Main Outcomes in CRASH-2

We specified the primary outcome measure for CRASH-2 as death in hospital within 4 weeks of injury. Secondary outcome measures are receipt of a blood transfusion, the number of blood units transfused, surgical intervention, and occurrence of vascular events (stroke, myocardial infarction, pulmonary embolism, deep vein thrombosis). A simple and widely practicable treatment that reduces blood loss following trauma might prevent thousands of premature trauma deaths each year and, secondly, could reduce exposure to the risks of blood transfusion. Blood is a scarce and expensive resource, and major concerns remain about the risk of transfusion-transmitted infection. Trauma is common in parts of the world where the safety of blood transfusion is not ensured. A study in Uganda estimated the population-attributable fraction of HIV acquisition as a result of blood transfusion to be about 2%, although some estimates are much higher [9]. Full details of the data collection instruments are available at http://www.crash2.lshtm.ac.uk. In-hospital deaths, transfusion requirements, and any complications are recorded on a single-sided outcome form that is completed entirely from the hospital notes. No special trial-specific investigations are required.

## Trial Progress

**Figure 1:**
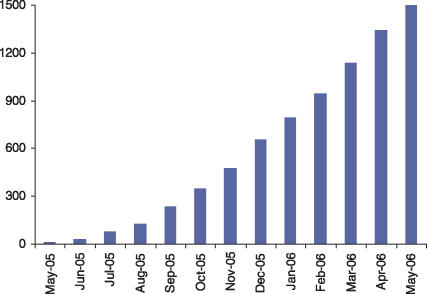
Randomisation Rate for the First 1,500 Patients

The recruitment during the first year of the trial is shown in Figure 1. The first patient was enrolled in May 2005, and, since then, more than 2,000 patients have been recruited at 60 participating hospitals, with a monthly recruitment rate of 200 patients per month. A further 60 hospitals have ethics committee approval, and many more have applied to take part and are seeking ethics committee approval. It is expected that the remaining 18,000 patients (2,000 recruited during the pilot phase) will be recruited over three years, with six months for data analysis, report preparation, and dissemination. The data-monitoring committee convened on 3 August 2006 and reviewed data for more than 1,800 randomised patients. The committee commended the collaborators on the excellent recruitment, and it was the view of the committee that “the interim analyses provided no reason for modifying the CRASH-2 protocol on the basis of safety or efficacy.”

Table 1 shows the characteristics of the first 1,500 randomised patients. As expected, there were more males (1,291, 86%) than females (209, 14%). The average age was 33 years (SD = 14, median = 30). Just less than half of the patients (49%) had blunt injury, 28% had penetrating injury, and 23% had both types. The median systolic blood pressure was 90 mm Hg. The majority of patients (70%) were randomised within three hours after injury.

**Table 1:**
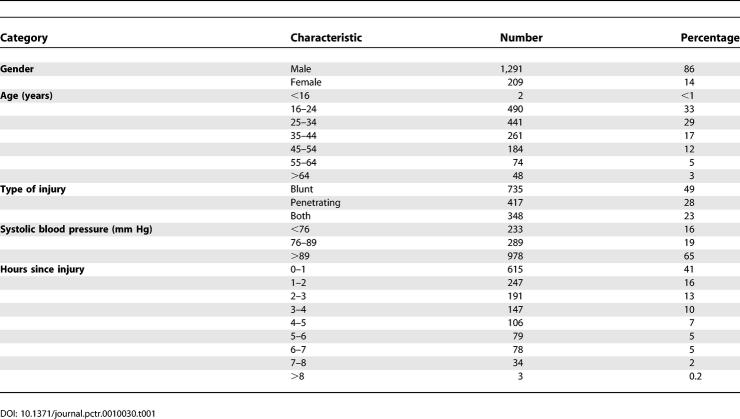
Baseline Characteristics

## Outcomes to Date

By the end of May 2006, outcomes from 1,268 randomized patients had been received. The outcome data included in-hospital deaths, transfusion requirements, number of units transfused, surgical intervention, and occurrence of thromboembolic episodes. The outcomes for this cohort of patients are shown in Table 2. The overall mortality rate was 19%, which was very close to that anticipated in the sample size estimates (20%). More than half of the patients (59%) received a blood transfusion. The prevalence of thromboembolic complications was low (less than 1%). Treatment compliance was determined on the basis that the entire treatment regimen was given. All randomised patients received the loading dose, and 93% received the maintenance (7% of patients died before the maintenance dose was completed).

**Table 2:**
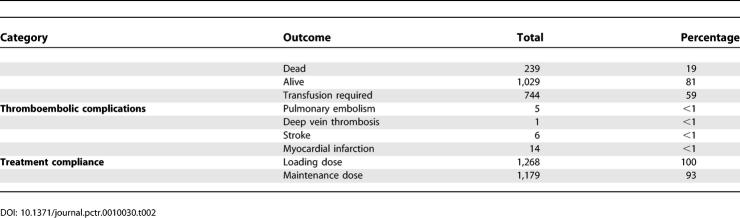
Summary of Outcome

## Comment on the CRASH-2 Experience: Our Perspective on Trauma Trials

Compared with the disease burden, there is a dearth of clinical trials in trauma care and the existing trials are small, contributing to uncertainty about effectiveness [10,11]. A major factor contributing to the lack of clinical trial evidence is the fact that funding for trauma research is less than for almost any other cause of human suffering.

Randomised controlled trials provide strong evidence that antifibrinolytics are effective in reducing blood loss during elective surgery, but to date there has been only one small trial of the use of antifibrinolytics in reducing blood loss after trauma. We intend that the CRASH-2 trial will assess reliably the effect of the early administration of the antifibrinolytic TXA on mortality and transfusion requirements in adult trauma patients with significant haemorrhage.

Following the successful completion of the first CRASH trial funded by the UK Medical Research Council, there is a well-established trial-coordinating team and a network of some 400 collaborating hospitals, many of which are now recruiting patients into the CRASH-2 trial and others that will start recruiting in the coming months. Progress to date shows that the trial procedures work efficiently, that baseline event rates are similar to those anticipated, and that physicians around the world are very interested in participating in this trial.

The trial collaborators have made an outstanding contribution by ensuring that CRASH-2 is already the largest clinical trial in traumatic haemorrhage. However, in order to achieve the CRASH-2 trial objectives, many more participating hospitals are required. We invite physicians from around the world to join this international collaboration and, in this way, to help build the evidence base for trauma care.
